# Fatty acid translocase: A culprit of lipid metabolism dysfunction in disease

**DOI:** 10.1097/IN9.0000000000000001

**Published:** 2022-08-15

**Authors:** Joseph E. Rupert, Mikhail G. Kolonin

**Affiliations:** The Institute of Molecular Medicine for the Prevention of Human Disease, The University of Texas Health Science Center at Houston, 7000 Fannin Street, Houston, Texas 77030, USA

**Keywords:** fatty acid translocase, lipid metabolism

## Abstract

Dysregulation of lipid deposition into and mobilization from white adipose tissue (WAT) underlies various diseases. Long-chain fatty acids (LCFA) and cholesterol trafficking in and out of adipocytes is a process relying on transporters shuttling lipids from the plasma membrane (PM) to lipid droplets (LD). CD36 is the fatty acid translocase (FAT) that transports LCFA and cholesterol across the PM. Interactions of CD36 with proteins PHB1, ANX2, and CAV1 mediate intercellular lipid transport between adipocytes, hematopoietic, epithelial, and endothelial cells. Intracellularly, the FAT complex has been found to regulate LCFA trafficking between the PM and LD. This process is regulated by CD36 glycosylation and *S*-acylation, as well as by post-translational modifications of PHB1 and ANX2, which determine both protein-protein interactions and the cellular localization of the complex. Changes in extracellular and intracellular LCFA levels have been found to induce the post-translational modifications and the function of the FAT complex in lipid uptake and mobilization. The role of the CD36/PHB1/ANX2 complex may span beyond lipid trafficking. The requirement of PHB1 for mitochondrial oxidative metabolism in brown adipocytes has been revealed. Cancer cells, which take advantage of lipids mobilized by adipocytes and oxidized in leukocytes are indirectly affected by the function of FAT complex in other tissues. The direct importance of CD36 interaction with PHB1 /and ANX2 in cancer cells remains to be established. This review highlights the multifaceted roles of the FAT complex in systemic lipid trafficking and discuss it as a potential target in metabolic disease and cancer.

## 1. The Role of CD36 in health and disease

Changes in lipid metabolism underlie the progression of all diseases ^[1]^. Fatty acids (FA), cholesterol, their derivatives, and other lipids are used as a source of energy, cell structure components, and signaling molecules. White adipocytes are the rheostat of FA, contained as triglycerides in the lipid droplets (LD), as well as of cholesterol. White adipose tissue (WAT) can store or release lipids, which affects metabolism and the immune system systemically ^[2,3]^. In contrast, brown adipose tissue (BAT) and beige adipocytes in WAT serve as energy sink by catabolizing lipids through adaptive thermogenesis ^[4]^. Adipocyte dysfunction and imbalance between lipid storage and mobilization aggravates chronic cardiovascular, inflammatory and malignant conditions ^[5]^. Adipocyte hypertrophy, a hallmark of obesity, leads to WAT inflammation, dyslipidemia, and systemic lipotoxicity resulting in type-2 diabetes ^[6,7]^. Conversely, excessive WAT lipolysis and lipid mobilization is associated with cachexia and cancer progression ^[8–10]^. The transport of lipids across the PM is an active process controlling cell metabolism. A key mediator of lipid transport is the scavenger receptor cluster of differentiation 36 (CD36), also known as fatty acid translocase (FAT). This protein, along with co-factors such as Src family kinases and G protein-coupled receptors (GPCRs), facilitates the intracellular and extracellular trafficking of long-chain fatty acids (LCFA), cholesterol, oxidized low-density lipoprotein (OxLDL) and extracellular matrix proteins ^[11–15]^. The interaction of CD36 with Src kinases and GPCRs allows for detection of specific lipids and the downstream activation of unique signaling pathways. For instance, CD36 binding of oleic acid activates mTOR and ERK1/2 pathways and is associated with tumor cell progression and metastasis ^[16,17]^. While CD36 interaction with GPR120 and GPR40 in tastebuds is implicated in the perception of dietary lipids and may play a role in obesity-associated diseases ^[18]^.

Ample evidence indicates the importance of CD36 in AT. CD36 inhibition attenuates the effect of lipolysis induction ^[19]^ and reduces hypertrophy of visceral WAT in mice fed high-fat diet ^[20,21]^. Based on this notion, CD36 has been investigated as a therapeutic target. However, these pursuits have been complicated because CD36 is expressed by various cell types including hematopoietic ^[22]^, and epithelial and endothelial cells ^[23,24]^ in which it plays diverse roles. In models of atherosclerosis, suppression of CD36 transcription ^[25,26]^, function ^[27]^, or increased CD36 degradation ^[28]^ were associated with a reduction in foam cell formation and reduced uptake of oxidized lipids by macrophages. Consistent with a pathogenic role of CD36, its inhibition attenuated hepatic steatosis/fibrosis ^[29,30]^ and myosteatosis ^[31]^. However, global deletion of CD36 results in dyslipidemia, subclinical inflammation ^[32]^ and an increased risk of atherosclerosis ^[33]^. Moreover, expression of CD36 was found to be an important regulator of proper muscle satellite cell differentiation and myofiber regeneration ^[34]^. Expression of CD36 is also important for suppressing neuroinflammation in demyelinating disorders ^[35]^. Collectively, these findings suggest that targeting the CD36 pathway requires cell type-specific strategies, and systemic inhibition of CD36 may have undesirable off-target effects.

## 2. Formation of the PHB1/ANX2/CD36 complex and its Importance in lipid trafficking and mitochondrial homeostasis

The complexity of FAT signaling, and its functions has become even more apparent since the identification of posttranslational modifications regulating CD36-induced lipid trafficking from the plasma membrane (PM) to the LD and vice versa. Glycosylation is one such modification, which is particularly pronounced in adipocytes ^[36,37]^. Another modification is *S*-acylation of CD36 by the palmitoyl acyltransferases DHHC4 or DHHC5. Palmitoylation induces CD36 translocation to the PM, which enables its binding of extracellular lipids and the subsequent internalization of the lipid-FAT complex via caveolin-1 (CAV1)-mediated endocytosis ^[38]^. Other proteins mediating this process are Prohibitin-1 (PHB1) and Annexin 2 (ANX2), which interact with CD36 at the surface of adipose endothelium and adipocytes ^[39]^. Dynamic *S*-acylation of PHB1 and ANX2 is important for the formation of the PHB1/ANX2/CD36 complex and its trafficking to and from the PM ^[40]^. Furthermore, lipolysis induction results in CD36 deacylation, internalization, and an apparent dissociation from PHB1 and ANX2 in the cytosol. Transient colocalization of CD36 and CAV1 at the LD during LCFA transport suggests that CD36 may play a role in lipid trafficking not only at the cell surface but also for transporting lipids from the PM to the LD. The detailed roles of PHB1 and ANX2 in regulating CD36-mediated lipid transport remain to be completely understood. While the inhibition of either PHB1 or ANX2 leads to a phenotype akin to CD36 knockout and ^[39,41]^, these proteins have other functions in addition to lipid transport. Both PHB1 and ANX2 are found in various cell organelles, in which they regulate distinct processes ^[41–48]^. A key function of PHB1 is to maintain mitochondrial biogenesis and function ^[49–51]^. Indeed, ablation of PHB1 in adipocytes results in decreased mitochondrial content and function, which accounts for the loss of BAT and thermogenesis ^[52,53]^. Interestingly, PHB1 does not possess a mitochondrial targeting domain, instead, phosphorylation of PHB1 at Thr258 by AKT is considered the mechanism promoting mitochondrial localization of PHB1 ^[54]^.

Since the discovery of the interaction of PHB1 with CD36 in adipocytes, and the critical role of PHB1 in mitochondrial respiration, it is logical to extrapolate this mechanism to non-adipogenic cells and postulate that the PHB1/ANX2/CD36/CAV1 interactome may act as a ubiquitous lipid-sensing pathway. One possibility is that lipid delivery to the mitochondrial membrane and stabilization of electron transport chain enzymes, mediated by these proteins, may increase mitochondrial oxidative phosphorylation and biogenesis, favoring FA oxidation for ATP generation. This concept could have critical implications in the context of tumor cell physiology. Tumor cells regularly endure hypoxia, changes in fuel source availability, and high levels of reactive oxygen species (ROS). These processes involve changes in mitochondrial function that are likely to engage PHB1 and hence relay back to the FAT complex.

As previously stated, CD36 has been shown to regulate mTOR signaling yet our understanding of the mechanism by which this occurs remains lacking. Interestingly, PHB1 plays a direct role in the regulation of mTOR signaling. While a consensus suggests PHB1 increases protein translation via activation of mTOR signaling ^[55,56]^, some studies report contradictory findings ^[57]^. Given its association with CD36 and status as a chaperone protein ^[50]^, PHB1 may act as a rheostat for FA metabolism in cells, regulating the shuttling of CD36 between the PM and LD while also activating mTOR and stabilizing mitochondrial metabolism and ATP production. ANX2 binds calcium, actin, and lipids and is a crucial component for stabilizing and organizing lipid microdomains at the PM, which are important sites for endo- and exocytosis ^[58–60]^. Thus, ANX2 may have a role in directing CD36 to specific sites at the PM for lipid trafficking. These implied roles for PHB1 and ANX2 in CD36-mediated FA transport warrant investigation to increase our understanding of these interactions.

## 3. CD36 and cancer

The importance of lipid metabolism in cancer has been realized in recent years ^[61–63]^. Progression of carcinomas to epithelial-to-mesenchymal (EMT), chemotherapy resistance, and metastasis is linked with increased LCFA uptake by tumors ^[64,65]^. Recent studies of our group demonstrated the role of adipocyte and endothelial CD36 in LCFA mobilization from WAT and their bioavailability for cancer cells ^[40,63]^. However, CD36 is also expressed by cancer cells, in which its glycosylation is relatively low, and the function is debated ^[40]^. Increased expression of CD36 in tumor epithelium is associated with poor prognosis of various GI carcinomas as well as of ovarian cancer, glioblastoma, oral squamous cell carcinoma, and melanoma ^[66]^. Interestingly, it is low CD36 expression that marks a poor prognosis for kidney renal clear cell carcinoma and pancreatic adenocarcinoma ^[67]^. Conflicting results have been published for breast cancer ^[68,69]^. Despite this controversy, there appears to be a clear link of high CD36 expression, particularly in combination with CAV1 expression, and metastasis ^[70]^. Pharmacological intervention results are consistent with cell surface CD36 being an important promoter of cancer aggressiveness ^[71]^. Studies have identified CD36 expression as a driver of EMT in cervical ^[72]^, ovarian ^[73]^, colon ^[74]^, breast ^[75]^, hepatocellular carcinoma ^[76]^, and pancreatic ^[77]^ cancers.

CD36 expression in the tumor microenvironment is also a key component of tumor progression. It has been reported that CD36-mediated uptake of oxidized lipids by leukocytes undermines anti-tumor immune response ^[66,78,79]^. Increased CD36-mediated uptake of oxidized lipids by killer T-cells causes them to become dysfunctional and switch from a cytotoxic to immunosuppressive role ^[79]^. Studies investigating tumor-associated macrophages (TAMs) show that blocking CD36-mediated uptake of oxidized lipids by TAMs significantly reduces tumor progression by reducing pro-tumor cytokines produced from TAMs ^[80]^. Interestingly, in breast cancer down-regulation of CD36 expression in cancer-associated fibroblasts is linked with reduced tumor cell proliferation ^[81]^. The CD36-interacting proteins have been individually implicated in promoting cancer progression. Elevated PHB1 is associated with increased metastasis in lung ^[82]^ and prostate ^[83]^ cancers, while both PHB1 and ANX2 have been implicated in breast cancer metastasis ^[84,85]^. A possible role of the PHB1/ANX2/CD36/CAV1 interactome in cancer progression remains to be determined and put into the context of what is known about other proteins, such as FATP1, which also regulate fatty acid transport in cancer cells ^[86]^.

## 4. Targeting CD36 as a novel therapy for metabolic and metastatic disease

Despite the paucity of the FAT complex functions, its directed targeting may still pose an attractive approach for treating metabolic diseases and warrants further investigation. Clinical studies targeting CD36 signaling in cancer are underway ^[66]^. Those that have been completed have largely failed due to off-target and adverse side effects, suggesting a refined approach is needed to increase the accuracy of inhibiting CD36 in target tissues ^[87,88]^. Our group has used homing peptides to direct experimental therapies to specific cell types ^[89–93]^. In brief, this approach utilizes combinatorial phage-displayed peptide libraries to identify peptide sequences for homing affinity to target cells ^[94]^. After screening, candidate peptides are validated for receptor-blocking properties. For example, a peptide mimicking the PHB1-binding site in ANX2 has been shown to block FA transport ^[39]^. Potentially, receptor-homing peptides could be used to deliver molecules neutralizing CD36 complex transport function and signaling specifically in WAT, hepatocytes, or other cells for the treatment of obesity, hepatosteatosis, type-2 diabetes, and metastatic disease. Furthermore, specific targeting of CD36 in cancer cells using phage-display may be an approach to interfere with tumor lipid metabolism, hence making chemotherapy more effective. However, the notion that the loss of PHB1 in adipocytes and in endothelial cells has opposite effects on metabolism ^[95]^ reiterates the point that targeting molecules in the FAT complex may need to be directed to specific cell types in order to be beneficial.

Increased interest in the relevance of lipid trafficking in disease will undoubtedly advance our understanding of metabolic and malignant disease. However, several aspects of what role the PHB1/ANX2/CD36 complex plays requires further investigation. For instance, CD36 has been shown to bind various ligands resulting in differential activation of downstream pathways, especially regarding Src family kinases and GPCRs. Detailed mechanisms showing how different classes of lipids binding the PHB1/ANX2/CD36 complex alters its interaction with secondary messengers, including any conformational changes, post-translational modification sites, and protein-protein interactions needs to be elucidated. Furthermore, the premise that this complex may act as a lipid-sensing pathway affecting intracellular lipid trafficking and metabolism in multiple cell types is intriguing. Detailed analyses of the functions of PHB1 and ANX2 after dissociation from the complex, especially if novel roles are identified for their involvement in lipid metabolism, protein synthesis, and mitochondrial function, would be important advances for linking lipids with chemoresistance and cancer progression. Future studies will establish and build on the relative importance of the FAT complex proteins in cells of WAT and BAT, versus other benign and malignant tissues.

## References

[1] Rosen ED, Spiegelman BM. What we talk about when we talk about fat. Cell. 2014;156(1–2):20–44. 24439368 10.1016/j.cell.2013.12.012 PMC3934003

[2] de Heredia FP, Gomez-Martinez S, Marcos A. Obesity, inflammation and the immune system. Proc Nutr Soc. 2012;71(2):332–8. 22429824 10.1017/S0029665112000092

[3] Yazici D, Sezer H. Insulin Resistance, Obesity and Lipotoxicity. Adv Exp Med Biol. 2017;960:277–304. 28585204 10.1007/978-3-319-48382-5_12

[4] Kajimura S, Spiegelman BM, Seale P. Brown and Beige Fat: Physiological Roles beyond Heat Generation. Cell Metab. 2015;22(4):546–59. 26445512 10.1016/j.cmet.2015.09.007 PMC4613812

[5] Morigny P, Boucher J, Arner P, Langin D. Lipid and glucose metabolism in white adipocytes: pathways, dysfunction and therapeutics. Nat Rev Endocrinol. 2021;17(5):276–95. 33627836 10.1038/s41574-021-00471-8

[6] Goossens GH. The Metabolic Phenotype in Obesity: Fat Mass, Body Fat Distribution, and Adipose Tissue Function. Obes Facts. 2017;10(3):207–15. 28564650 10.1159/000471488 PMC5644968

[7] Lytrivi M, Castell AL, Poitout V, Cnop M. Recent Insights Into Mechanisms of beta-Cell Lipo- and Glucolipotoxicity in Type 2 Diabetes. J Mol Biol. 2020;432(5):1514–34. 31628942 10.1016/j.jmb.2019.09.016 PMC7073302

[8] Rupert JE, Narasimhan A, Jengelley DHA, Tumor-derived IL-6 and trans-signaling among tumor, fat, and muscle mediate pancreatic cancer cachexia. J Exp Med. 2021;218(6):e20190450. 33851955 10.1084/jem.20190450 PMC8185651

[9] Quail DF, Dannenberg AJ. The obese adipose tissue microenvironment in cancer development and progression. Nat Rev Endocrinol. 2019;15(3):139–54. 30459447 10.1038/s41574-018-0126-x PMC6374176

[10] Bacci M, Lorito N, Smiriglia A, Morandi A. Fat and Furious: Lipid Metabolism in Antitumoral Therapy Response and Resistance. Trends Cancer. 2021;7(3):198–213. 33281098 10.1016/j.trecan.2020.10.004

[11] Pepino MY, Kuda O, Samovski D, Abumrad NA. Structure-function of CD36 and importance of fatty acid signal transduction in fat metabolism. Annu Rev Nutr. 2014;34:281–303. 24850384 10.1146/annurev-nutr-071812-161220 PMC4329921

[12] Glatz JF, Luiken JJ, Bonen A. Membrane fatty acid transporters as regulators of lipid metabolism: implications for metabolic disease. Physiol Rev. 2010;90(1):367–417. 20086080 10.1152/physrev.00003.2009

[13] Canton J, Neculai D, Grinstein S. Scavenger receptors in homeostasis and immunity. Nat Rev Immunol. 2013;13(9):621–34. 23928573 10.1038/nri3515

[14] Silverstein RL, Febbraio M. CD36, a scavenger receptor involved in immunity, metabolism, angiogenesis, and behavior. Sci Signal. 2009;2(72):re3. 19471024 10.1126/scisignal.272re3 PMC2811062

[15] Asch AS, Silbiger S, Heimer E, Nachman RL. Thrombospondin sequence motif (CSVTCG) is responsible for CD36 binding. Biochem Biophys Res Commun. 1992;182(3):1208–17. 1371676 10.1016/0006-291x(92)91860-s

[16] Yang P, Su C, Luo X, Dietary oleic acid-induced CD36 promotes cervical cancer cell growth and metastasis via up-regulation Src/ERK pathway. Cancer Lett. 2018;438:76–85. 30213558 10.1016/j.canlet.2018.09.006

[17] Luo X, Zheng E, Wei L, The fatty acid receptor CD36 promotes HCC progression through activating Src/PI3K/AKT axis-dependent aerobic glycolysis. Cell Death Dis. 2021;12(4):328. 33771982 10.1038/s41419-021-03596-w PMC7997878

[18] Gilbertson TA, Khan NA. Cell signaling mechanisms of oro-gustatory detection of dietary fat: advances and challenges. Prog Lipid Res. 2014;53:82–92. 24269201 10.1016/j.plipres.2013.11.001

[19] Zhou D, Samovski D, Okunade AL, Stahl PD, Abumrad NA, Su X. CD36 level and trafficking are determinants of lipolysis in adipocytes. FASEB J. 2012;26(11):4733–42. 22815385 10.1096/fj.12-206862 PMC3475246

[20] Su T, Huang C, Yang C, Apigenin inhibits STAT3/CD36 signaling axis and reduces visceral obesity. Pharmacol Res. 2020;152:104586. 31877350 10.1016/j.phrs.2019.104586

[21] Yang J, Park KW, Cho S. Inhibition of the CD36 receptor reduces visceral fat accumulation and improves insulin resistance in obese mice carrying the BDNF-Val66Met variant. J Biol Chem. 2018;293(34):13338–48. 29914985 10.1074/jbc.RA118.002405 PMC6109932

[22] Yang M, Silverstein RL. CD36 signaling in vascular redox stress. Free Radic Biol Med. 2019;136:159–71. 30825500 10.1016/j.freeradbiomed.2019.02.021 PMC6488418

[23] Cifarelli V, Appak-Baskoy S, Peche VS, Visceral obesity and insulin resistance associate with CD36 deletion in lymphatic endothelial cells. Nat Commun. 2021;12(1):3350. 34099721 10.1038/s41467-021-23808-3 PMC8184948

[24] Febbraio M, Hajjar DP, Silverstein RL. CD36: a class B scavenger receptor involved in angiogenesis, atherosclerosis, inflammation, and lipid metabolism. J Clin Invest. 2001;108(6):785–91. 11560944 10.1172/JCI14006 PMC200943

[25] Ackers I, Szymanski C, Duckett KJ, Consitt LA, Silver MJ, Malgor R. Blocking Wnt5a signaling decreases CD36 expression and foam cell formation in atherosclerosis. Cardiovasc Pathol. 2018;34:1–8. 29474941 10.1016/j.carpath.2018.01.008

[26] Jia Q, Cao H, Shen D, Quercetin protects against atherosclerosis by regulating the expression of PCSK9, CD36, PPARγ, LXRα and ABCA1. Int J Mol Med. 2019;44(3):893–902. 31524223 10.3892/ijmm.2019.4263 PMC6658003

[27] Fan Y, Yang J, Li H, SNX10 deficiency restricts foam cell formation and protects against atherosclerosis by suppressing CD36-Lyn axis. Can J Cardiol. 2020. doi: 10.1016/j.cjca.2020.05.010 32428616

[28] Xia X, Hu T, He J, USP10 deletion inhibits macrophage-derived foam cell formation and cellular-oxidized low density lipoprotein uptake by promoting the degradation of CD36. Aging. 2020;12(22):22892–905. 33197885 10.18632/aging.104003 PMC7746336

[29] Lin HY, Wang FS, Yang YL, Huang YH. MicroRNA-29a Suppresses CD36 to Ameliorate High Fat Diet-Induced Steatohepatitis and Liver Fibrosis in Mice. Cells. 2019;8(10):1298. 10.3390/cells8101298 PMC6830328 31652636

[30] Zhang C, Luo X, Chen J, Osteoprotegerin Promotes Liver Steatosis by Targeting the ERK-PPAR-γ-CD36 Pathway. Diabetes. 2019;68(10):1902–14. 31292134 10.2337/db18-1055

[31] Glatz JFC, Luiken JJFP. Dynamic role of the transmembrane glycoprotein CD36 (SR-B2) in cellular fatty acid uptake and utilization. J Lipid Res. 2018;59(7):1084–93. 29627764 10.1194/jlr.R082933 PMC6027920

[32] Cifarelli V, Ivanov S, Xie Y, CD36 deficiency impairs the small intestinal barrier and induces subclinical inflammation in mice. Cell Mol Gastroenterol Hepatol. 2017;3(1):82–98. 28066800 10.1016/j.jcmgh.2016.09.001 PMC5217470

[33] Zhao L, Varghese Z, Moorhead JF, Chen Y, Ruan XZ. CD36 and lipid metabolism in the evolution of atherosclerosis. Br Med Bull. 2018;126(1):101–12. 29534172 10.1093/bmb/ldy006

[34] Verpoorten S, Sfyri P, Scully D, Loss of CD36 protects against diet-induced obesity but results in impaired muscle stem cell function, delayed muscle regeneration and hepatic steatosis. Acta Physiol. 2020;228(3):e13395. 10.1111/apha.13395 31599493

[35] Grajchen E, Wouters E, van de Haterd B, CD36-mediated uptake of myelin debris by macrophages and microglia reduces neuroinflammation. J Neuroinflammation. 2020;17(1):224. 32718316 10.1186/s12974-020-01899-x PMC7384221

[36] Hoosdally SJ, Andress EJ, Wooding C, Martin CA, Linton KJ. The Human Scavenger Receptor CD36: glycosylation status and its role in trafficking and function. J Biol Chem. 2009;284(24):16277–88. 19369259 10.1074/jbc.M109.007849 PMC2713513

[37] Abumrad NA, el-Maghrabi MR, Amri EZ, Lopez E, Grimaldi PA. Cloning of a rat adipocyte membrane protein implicated in binding or transport of long-chain fatty acids that is induced during preadipocyte differentiation. Homology with human CD36. J Biol Chem. 1993;268(24):17665–8. 7688729

[38] Wang J, Hao JW, Wang X, DHHC4 and DHHC5 Facilitate Fatty Acid Uptake by Palmitoylating and Targeting CD36 to the Plasma Membrane. Cell Rep. 2019;26(1):209–21.e5. 30605677 10.1016/j.celrep.2018.12.022

[39] Salameh A, Daquinag AC, Staquicini DI, Prohibitin/annexin 2 interaction regulates fatty acid transport in adipose tissue. JCI Insight. 2016;1(10):e86351. 27468426 10.1172/jci.insight.86351 PMC4959783

[40] Daquinag AC, Gao Z, Fussell C, Fatty acid mobilization from adipose tissue is mediated by CD36 posttranslational modifications and intracellular trafficking. JCI Insight. 2021;6(17):e147057. 10.1172/jci.insight.147057 PMC8492349 34314388

[41] Kathiria AS, Butcher LD, Feagins LA, Souza RF, Boland CR, Theiss AL. Prohibitin 1 modulates mitochondrial stress-related autophagy in human colonic epithelial cells. PLoS One. 2012;7(2):e31231. 22363587 10.1371/journal.pone.0031231 PMC3281932

[42] Theiss AL, Idell RD, Srinivasan S, Prohibitin protects against oxidative stress in intestinal epithelial cells. FASEB J. 2007;21(1):197–206. 17135366 10.1096/fj.06-6801com

[43] Mielenz D, Vettermann C, Hampel M, Lipid rafts associate with intracellular B cell receptors and exhibit a B cell stage-specific protein composition. J Immunol. 2005;174(6):3508–17. 15749887 10.4049/jimmunol.174.6.3508

[44] Wang S, Fusaro G, Padmanabhan J, Chellappan SP. Prohibitin co-localizes with Rb in the nucleus and recruits N-CoR and HDAC1 for transcriptional repression. Oncogene. 2002;21(55):8388–96. 12466959 10.1038/sj.onc.1205944

[45] Kube E, Becker T, Weber K, Gerke V. Protein-protein interaction studied by site-directed mutagenesis. Characterization of the annexin II-binding site on p11, a member of the S100 protein family. J Biol Chem. 1992;267(20):14175–82. 1385811

[46] Johnsson N, Weber K. Alkylation of cysteine 82 of p11 abolishes the complex formation with the tyrosine-protein kinase substrate p36 (annexin 2, calpactin 1, lipocortin 2). J Biol Chem. 1990;265(24):14464–8. 2143760

[47] Waisman DM. Annexin II tetramer: structure and function. Mol Cell Biochem. 1995;149–50:301–22. 10.1007/BF01076592 8569746

[48] Yurugi H, Tanida S, Ishida A, Expression of prohibitins on the surface of activated T cells. Biochem Biophys Res Commun. 2012;420(2):275–80. 22417827 10.1016/j.bbrc.2012.02.149

[49] Jackson DN, Panopoulos M, Neumann WL, Mitochondrial dysfunction during loss of prohibitin 1 triggers Paneth cell defects and ileitis. Gut. 2020;69(11):1928–38. 32111635 10.1136/gutjnl-2019-319523 PMC7483170

[50] Nijtmans LG, de Jong L, Artal Sanz M, Prohibitins act as a membrane-bound chaperone for the stabilization of mitochondrial proteins. EMBO J. 2000;19(11):2444–51. 10835343 10.1093/emboj/19.11.2444 PMC212747

[51] Xu Y, Shen J, Ran Z. Emerging views of mitophagy in immunity and autoimmune diseases. Autophagy. 2020;16(1):3–17. 30951392 10.1080/15548627.2019.1603547 PMC6984455

[52] Liu D, Lin Y, Kang T, Mitochondrial dysfunction and adipogenic reduction by prohibitin silencing in 3T3-L1 cells. PLoS One. 2012;7(3):e34315. 22479600 10.1371/journal.pone.0034315 PMC3316679

[53] Gao Z, Daquinag AC, Fussell C, Djehal A, Désaubry L, Kolonin MG. Prohibitin Inactivation in Adipocytes Results in Reduced Lipid Metabolism and Adaptive Thermogenesis Impairment. Diabetes. 2021;70(10):2204–12. 34257070 10.2337/db21-0094 PMC8576510

[54] Jiang L, Dong P, Zhang Z, Akt phosphorylates Prohibitin 1 to mediate its mitochondrial localization and promote proliferation of bladder cancer cells. Cell Death Dis. 2015;6:e1660. 25719244 10.1038/cddis.2015.40 PMC4669803

[55] Zhang J, Yin Y, Wang J, Prohibitin regulates mTOR pathway via interaction with FKBP8. Front Med. 2021;15(3):448–59. 33259040 10.1007/s11684-020-0805-6

[56] Jin X, Xie J, Zabolocki M, The prohibitin-binding compound fluorizoline affects multiple components of the translational machinery and inhibits protein synthesis. J Biol Chem. 2020;295(29):9855–67. 32430400 10.1074/jbc.RA120.012979 PMC7380185

[57] Ising C, Brinkkoetter PT. Prohibitin Signaling at the Kidney Filtration Barrier. Adv Exp Med Biol. 2017;982:563–75. 28551807 10.1007/978-3-319-55330-6_29

[58] Bharadwaj A, Bydoun M, Holloway R, Waisman D. Annexin A2 heterotetramer: structure and function. Int J Mol Sci. 2013;14(3):6259–305. 23519104 10.3390/ijms14036259 PMC3634455

[59] Gabel M, Chasserot-Golaz S. Annexin A2, an essential partner of the exocytotic process in chromaffin cells. J Neurochem. 2016;137(6):890–6. 27037794 10.1111/jnc.13628

[60] Gabel M, Delavoie F, Demais V, Annexin A2-dependent actin bundling promotes secretory granule docking to the plasma membrane and exocytosis. J Cell Biol. 2015;210(5):785–800. 26323692 10.1083/jcb.201412030 PMC4555831

[61] Maan M, Peters JM, Dutta M, Patterson AD. Lipid metabolism and lipophagy in cancer. Biochem Biophys Res Commun. 2018;504(3):582–9. 29438712 10.1016/j.bbrc.2018.02.097 PMC6086774

[62] Snaebjornsson MT, Janaki-Raman S, Schulze A. Greasing the Wheels of the Cancer Machine: The Role of Lipid Metabolism in Cancer. Cell Metab. 2020;31(1):62–76. 31813823 10.1016/j.cmet.2019.11.010

[63] Lengyel E, Makowski L, DiGiovanni J, Kolonin MG. Cancer as a Matter of Fat: The Crosstalk between Adipose Tissue and Tumors. Trends Cancer. 2018;4(5):374–84. 29709261 10.1016/j.trecan.2018.03.004 PMC5932630

[64] Zhong X, Yang Y, Li B, Downregulation of SLC27A6 by DNA Hypermethylation Promotes Proliferation but Suppresses Metastasis of Nasopharyngeal Carcinoma Through Modulating Lipid Metabolism. Front Oncol. 2021;11:780410. 35047398 10.3389/fonc.2021.780410 PMC8761909

[65] Wang R, Tao B, Fan Q, Fatty-acid receptor CD36 functions as a hydrogen sulfide-targeted receptor with its Cys333-Cys272 disulfide bond serving as a specific molecular switch to accelerate gastric cancer metastasis. EBioMedicine. 2019;45:108–23. 31262715 10.1016/j.ebiom.2019.06.037 PMC6642364

[66] Wang J, Li Y. CD36 tango in cancer: signaling pathways and functions. Theranostics. 2019;9(17):4893–908. 31410189 10.7150/thno.36037 PMC6691380

[67] Chen YJ, Liao WX, Huang SZ, Prognostic and immunological role of CD36: A pan-cancer analysis. J Cancer. 2021;12(16e):4762–73. 34234847 10.7150/jca.50502 PMC8247371

[68] Feng WW, Wilkins O, Bang S, CD36-Mediated Metabolic Rewiring of Breast Cancer Cells Promotes Resistance to HER2-Targeted Therapies. Cell Rep. 2019;29(11):3405–20.e5. 31825825 10.1016/j.celrep.2019.11.008 PMC6938262

[69] DeFilippis RA, Chang H, Dumont N, CD36 repression activates a multicellular stromal program shared by high mammographic density and tumor tissues. Cancer Discov. 2012;2(9):826–39. 22777768 10.1158/2159-8290.CD-12-0107 PMC3457705

[70] Nath A, Chan C. Genetic alterations in fatty acid transport and metabolism genes are associated with metastatic progression and poor prognosis of human cancers. Sci Rep. 2016;6:18669. 26725848 10.1038/srep18669 PMC4698658

[71] Pascual G, Avgustinova A, Mejetta S, Targeting metastasis-initiating cells through the fatty acid receptor CD36. Nature. 2017;541(7635):41–5. 27974793 10.1038/nature20791

[72] Deng M, Cai X, Long L, CD36 promotes the epithelial-mesenchymal transition and metastasis in cervical cancer by interacting with TGF-β. J Transl Med. 2019;17(1):352. 31655604 10.1186/s12967-019-2098-6 PMC6815430

[73] Ladanyi A, Mukherjee A, Kenny HA, Adipocyte-induced CD36 expression drives ovarian cancer progression and metastasis. Oncogene. 2018;37(17):2285–301. 29398710 10.1038/s41388-017-0093-z PMC5920730

[74] Corbet C, Bastien E, Santiago de Jesus JP, TGFβ2-induced formation of lipid droplets supports acidosis-driven EMT and the metastatic spreading of cancer cells. Nat Commun. 2020;11(1):454. 31974393 10.1038/s41467-019-14262-3 PMC6978517

[75] Gyamfi J, Yeo JH, Kwon D, Interaction between CD36 and FABP4 modulates adipocyte-induced fatty acid import and metabolism in breast cancer. NPJ Breast Cancer. 2021;7(1):129. 34561446 10.1038/s41523-021-00324-7 PMC8463699

[76] Nath A, Li I, Roberts LR, Chan C. Elevated free fatty acid uptake via CD36 promotes epithelial-mesenchymal transition in hepatocellular carcinoma. Sci Rep. 2015;5:14752. 26424075 10.1038/srep14752 PMC4589791

[77] Nan P, Dong X, Bai X, Tumor-stroma TGF-β1-THBS2 feedback circuit drives pancreatic ductal adenocarcinoma progression via integrin α. Cancer Lett. 2022;528:59–75. 34958892 10.1016/j.canlet.2021.12.025

[78] Wang H, Franco F, Tsui YC, CD36-mediated metabolic adaptation supports regulatory T cell survival and function in tumors. Nat Immunol. 2020;21(3):298–308. 32066953 10.1038/s41590-019-0589-5 PMC7043937

[79] Xu S, Chaudhary O, Rodríguez-Morales P, Uptake of oxidized lipids by the scavenger receptor CD36 promotes lipid peroxidation and dysfunction in CD8. Immunity. 2021;54(7):1561–77.e7. 34102100 10.1016/j.immuni.2021.05.003 PMC9273026

[80] Su P, Wang Q, Bi E, Enhanced Lipid Accumulation and Metabolism Are Required for the Differentiation and Activation of Tumor-Associated Macrophages. Cancer Res. 2020;80(7):1438–50. 32015091 10.1158/0008-5472.CAN-19-2994 PMC7127942

[81] Jabbari K, Winkelmaier G, Andersen C, Protein Ligands in the Secretome of CD36. Cancers. 2021;13(18):4521. 34572749 10.3390/cancers13184521 PMC8469330

[82] Ho MY, Liang CM, Liang SM. MIG-7 and phosphorylated prohibitin coordinately regulate lung cancer invasion/metastasis. Oncotarget. 2015;6(1):381–93. 25575814 10.18632/oncotarget.2804 PMC4381602

[83] Cho SY, Kang S, Kim DS, HSP27, ALDH6A1 and Prohibitin Act as a Trio-biomarker to Predict Survival in Late Metastatic Prostate Cancer. Anticancer Res. 2018;38(11):6551–60. 30396985 10.21873/anticanres.13021

[84] Huang X, Liu J, Ma Q. Prohibitin participates in the HIRA complex to promote cell metastasis in breast cancer cell lines. FEBS Open Bio. 2020;10(10):2182–90. 10.1002/2211-5463.12966 PMC7530387 32865342

[85] Maji S, Chaudhary P, Akopova I, Exosomal Annexin II Promotes Angiogenesis and Breast Cancer Metastasis. Mol Cancer Res. 2017;15(1):93–105. 27760843 10.1158/1541-7786.MCR-16-0163 PMC5215956

[86] Zhang M, Di Martino JS, Bowman RL, Adipocyte-Derived Lipids Mediate Melanoma Progression via FATP Proteins. Cancer Discov. 2018;8(8):1006–25. 29903879 10.1158/2159-8290.CD-17-1371 PMC6192670

[87] Jeanne A, Schneider C, Martiny L, Dedieu S. Original insights on thrombospondin-1-related antireceptor strategies in cancer. Front Pharmacol. 2015;6:252. 26578962 10.3389/fphar.2015.00252 PMC4625054

[88] Molckovsky A, Siu LL. First-in-class, first-in-human phase I results of targeted agents: highlights of the 2008 American society of clinical oncology meeting. J Hematol Oncol. 2008;1:20. 18959794 10.1186/1756-8722-1-20 PMC2647552

[89] Kolonin MG, Saha PK, Chan L, Pasqualini R, Arap W. Reversal of obesity by targeted ablation of adipose tissue. Nat Med. 2004;10(6):625–32. 15133506 10.1038/nm1048

[90] Daquinag AC, Zhang Y, Amaya-Manzanares F, Simmons PJ, Kolonin MG. An isoform of decorin is a resistin receptor on the surface of adipose progenitor cells. Cell Stem Cell. 2011;9(1):74–86. 21683670 10.1016/j.stem.2011.05.017

[91] Daquinag AC, Tseng C, Zhang Y, Targeted Proapoptotic Peptides Depleting Adipose Stromal Cells Inhibit Tumor Growth. Mol Ther. 2016;24(1):34–40. 26316391 10.1038/mt.2015.155 PMC4754543

[92] Daquinag AC, Tseng C, Salameh A, Depletion of white adipocyte progenitors induces beige adipocyte differentiation and suppresses obesity development. Cell Death Differ. 2015;22(2):351–63. 25342467 10.1038/cdd.2014.148 PMC4291494

[93] Subramanian S, Daquinag AC, AghaAmiri S, Ghosh SC, Azhdarinia A, Kolonin MG. Characterization of Peptides Targeting Metastatic Tumor Cells as Probes for Cancer Detection and Vehicles for Therapy Delivery. Cancer Res. 2021;81(22):5756–64. 34607842 10.1158/0008-5472.CAN-21-1015

[94] Wu CH, Liu IJ, Lu RM, Wu HC. Advancement and applications of peptide phage display technology in biomedical science. J Biomed Sci. 2016;23:8. 26786672 10.1186/s12929-016-0223-x PMC4717660

[95] Gao Z, Daquinag AC, Yu Y, Kolonin MG. Endothelial Prohibitin Mediates Bidirectional Long- Chain Fatty Acid Transport in White and Brown Adipose Tissues. Diabetes. 2022 May 2, doi: 10.2337/db21-0972 PMC9233243 35499627

